# Role of CD38 in anti-tumor immunity of small cell lung cancer

**DOI:** 10.3389/fimmu.2024.1348982

**Published:** 2024-03-12

**Authors:** Hirokazu Taniguchi, Shweta S. Chavan, Andrew Chow, Joseph M. Chan, Hiroshi Mukae, Charles M. Rudin, Triparna Sen

**Affiliations:** ^1^ Department of Respiratory Medicine, Nagasaki University Graduate School of Biomedical Sciences, Nagasaki, Japan; ^2^ Department of Medicine, Memorial Sloan Kettering Cancer Center, New York, NY, United States; ^3^ Marie-Josée and Henry R. Kravis Center for Molecular Oncology, Memorial Sloan Kettering Cancer Center, New York, NY, United States; ^4^ Department of Human Oncology and Pathogenesis Program, Memorial Sloan Kettering Cancer Center, New York, NY, United States; ^5^ Weill Cornell Medical College, New York, NY, United States; ^6^ Department of Oncological Sciences, Tisch Cancer Institute, Icahn School of Medicine at Mount Sinai, New York, NY, United States

**Keywords:** small cell lung cancer, CD38, immune checkpoint blockade, resistance, lung cancer

## Abstract

**Introduction:**

Immune checkpoint blockade (ICB) with or without chemotherapy has a very modest benefit in patients with small cell lung cancer (SCLC). SCLC tumors are characterized by high tumor mutation burden (TMB) and low PD-L1 expression. Therefore, TMB and PD-L1 do not serve as biomarkers of ICB response in SCLC. CD38, a transmembrane glycoprotein, mediates immunosuppression in non-small cell lung cancer (NSCLC). In this brief report, we highlight the potential role of CD38 as a probable biomarker of immunotherapy response in SCLC.

**Methods:**

We evaluated the role of CD38 as a determinant of tumor immune microenvironment in SCLC with bulk and single-cell transcriptomic analyses and protein assessments of clinical samples and preclinical models, including CD38 *in vivo* blockade.

**Results:**

In SCLC clinical samples, *CD38* levels were significantly correlated with the gene expression of the immunosuppressive markers *FOXP3*, *PD-1* and *CTLA-4*. CD38 expression was significantly enhanced after chemotherapy and ICB treatment in SCLC preclinical models and clinical samples. A combination of cisplatin/etoposide, ICB, and CD38 blockade delayed tumor growth compared to cisplatin/etoposide.

**Conclusion:**

Our study provides a preliminary but important direction toward exploring CD38 as a potential biomarker of ICB response and CD38 blockade as a combination strategy for chemo-immunotherapy in SCLC.

## Introduction

CD38 is a transmembrane glycoprotein, widely expressed on hematopoietic and non-hematopoietic cells (1) and has been recognized in the regulation of extracellular metabolites, cell adhesion, signal transduction, and intracellular Ca^2+^ signaling. Targeting CD38 with the anti-CD38 antibody daratumumab and isatuximab are FDA-approved for the treatment of patients with multiple myeloma. It has previously been reported that CD38-mediated immunosuppression is a mechanism of tumor cell escape from PD-1/PD-L1 blockade in non-small cell lung cancer (NSCLC) and co-inhibition of CD38 and PD-L1 improved antitumor immune response in preclinical models of NSCLC (2).

Immune checkpoint blockade (ICB) targeting the PD-1/PD-L1 axis or CTLA-4 has a very limited effect on patients with small cell lung cancer (SCLC), the most aggressive form of lung cancer. Despite the relatively high tumor mutation burden (TMB) (3, 4), SCLC is characterized by an immunosuppressed phenotype that merits further study.

In this study, we explored the landscape of CD38 in tumors in patients with SCLC and the effect of CD38 blockade and platinum chemotherapy treatment, with or without PD-L1 blockade in SCLC models. This is the first study investigating the role of CD38 in anti-tumor immunity of SCLC.

## Materials and methods

### Cell lines and cell cultures

Human SCLC derived cell lines obtained from the American Type Culture Collection (ATCC). Cell lines were maintained in Roswell Park Memorial Institute (RPMI) media supplemented with 10% fetal bovine serum, penicillin (100 U/mL), and streptomycin (50 g/mL) and incubated at 37˚C with 5% CO2. Cell lines were tested and authenticated by short tandem repeat profiling (DNA fingerprinting) and routinely tested for mycoplasma species before any experiments were performed. The GEMM-derived SCLC cell lines derived from a conditional triple-knockout model of SCLC, *Trp53-/-, p130-/-, Rb1-/-* (RPP).

### Analysis of RNA-seq data

Bulk RNA-Seq datasets sourced from Rudin et al. and George et al. respectively. Correlations between CD38 and immune markers within these datasets were calculated in R using Spearman rank correlation test, a non-parametric test to compute a coefficient (rho) to quantify the extent of statistical dependence between two variables, and a p-value for determining the significance of correlation, with a p-value of <0.05 being statistically significant. Gene expression matrices with log transformed TPM (transcripts per million) values were centered and scaled and used as input to generate heatmaps using R package ‘ComplexHeatmaps’.

### Flow cytometry

SCLC cells were incubated with anti-CD16/32 monoclonal antibody TruFCX to block nonspecific binding, and then stained (30 min) with anti-human CD45 (BV510, clone 2D1), CD8 (PerCP-Cy5.5, clone SK1, CD4 (Alexa Fluor 700, clone A161A1), CD38 antibody (PE-Cy7, clone; HIT2). Dead cells were excluded use 4’,6-diamidino-2-phenylindole (DAPI, 1µg/ml) staining. SCLC cells were acquired on a Cytek Aurora (Cytek, Bethesda, MD) flow cytometer and primary samples were acquired on a LSRII flow cytometer. Data was analyzed with FlowJo software (version 10.6, BD Biosciences, Franklin Lakes, NJ).

### Patient cohorts

Patients with LUAD or SCLC undergoing a surgical resection or tissue biopsy at Memorial Sloan Kettering Cancer Center (MSKCC) were identified and biospecimens collected prospectively for scRNA profiling from 2017 to 2019. All patients from whom biospecimens were obtained provided informed consent through an Institutional Review Board (IRB)-approved biospecimen collection and analysis protocol. And Fresh primary tumors, metastatic lesions and pleural/peritoneal/pericardial effusions were obtained from August 2018 to September 2021 for flowcytometry. with permission from the MSKCC IRB. Informed consent was collected from all patients enrolled in this study.

### Animal experiments

All animal experiments were approved by the Memorial Sloan Kettering Cancer Center (MSKCC) Animal Care and Use Committee. Cell suspensions (2×10^6^ RPP cells) were injected subcutaneously in a 1:1 mixture of PBS and Matrigel (#CB40234, Fisher) into the flanks of 6-week-old female B6129F1 mice obtained from TACONIC. Once mean tumor volumes reached 100-150 mm3, mice were randomized and treated with isotype IgG control, anti-PD-L1 antibody (clone 10F.9G2) (200µg/body, once weekly, intraperitoneal injection [IP], anti-CD38 antibody (clone NIMR-5) (300µg/body, once weekly, IP), or anti-CD38 antibody and anti-PD-L1 antibody. For experiment of chemotherapy with or without anti-CD38 antibody and anti-PD-L1 antibody, cisplatin (2mg/body, once weekly, IP, Accord Healthcare), etoposide (3mg/body, 3 of 7 days, IP, Accord Healthcare) and anti-PD-L1 antibody (200µg/body, once weekly, IP), anti-CD38 antibody (200µg/body, once weekly, IP) were injected. The treatment was started from day7 after cell implantation and finished day 44. Tumors were measured twice a week using calipers and their volumes were calculated as width^2^ x length x 0.5. Body weights were monitored twice a week.

### Western blotting

Protein extraction was done in previously described method. Briefly, cells were harvested and washed with ice cold PBS followed by re-suspending the pelleted cells and in ice-cold RIPA buffer (ThermoFisher, #89901) supplemented with protease and phosphatase inhibitors (ThermoFisher, #78446). After incubating for 1 hour on ice suspension were centrifuged at 14,000 rpm for 10 min in a refrigerated benchtop centrifuge (Eppendorf, #5340 R) to prepare the cell free protein extracts. Protein lysates were quantified using a micro BCA protein assay kit (Pierce, #23235). Protein samples were prepared and run on SDS-PAGE followed by wet-transferring proteins to 0.45 μm Immobilon-FL PVDF membrane (Millipore, #IPFL00010). Membranes were blocked with Pierce Starting Block (PBS) Blocking Buffer (Thermo Fisher) at room temperature for 1 hr and then incubated overnight with primary antibodies (CD38: R and D, AF4947, actin: Cell Singaling, # 4970)(1:1000) at 4°C. Incubation with horseradish peroxidase-linked secondary antibody was done in room temperature at a concentration of 1:10000 and the bands were detected using iBright Western Blot Imaging Systems (Thermo Fisher).

### RNA isolation

RNA was isolated using the Direct-zol RNA MiniPrep Kit (Zymo Research, cat# R2050) according to the manufacturer’s instructions. RNA concentrations were determined using a NanoDrop 2000 UV-Vis spectrophotometer (Thermo Fisher Scientific).

### Reverse transcription

Reverse transcription reactions were carried out using SuperScript III First-Strand Synthesis SuperMix (Invitrogen, cat#18080-400) according to the manufacturer’s instructions.

### Real-time PCR

Real-time PCR was done using SYBR Select Master Mix (Life Technologies, cat# 4472908) according to the manufacturer’s protocol. The primers were purchased from Sigma (USA); CD38: forward: 5’-GGTCCAAGTGATGCTCAATGGG-3’ and reverse: 5’-AGCTCCTTCGATGTCGTGCATC-3’, GAPDH: forward: AGGGGAGATTCAGTGTGGTG, and reverse: GGCCTCCAAGGAGTAAGACC. Triplicate PCR reactions were run on ABI (7500 Fast Real Time PCR System) according to the manufacturer’s instructions. The comparative Ct method using the average 2ΔΔCT value for each set of triplicates was used, and the average of the biological replicates was calculated. Negative controls were included for every primer set, and GAPDH was used as the positive control.

## Results

### CD38 gene expression is associated with key immune suppressive markers in SCLC

To explore the relationship between the expression of CD38 and potential immunosuppression in SCLC, we evaluated the expression of *CD38* and genes involved in anti-tumor immunity from two publicly available SCLC clinical datasets (5, 6).

**Figure 1:**
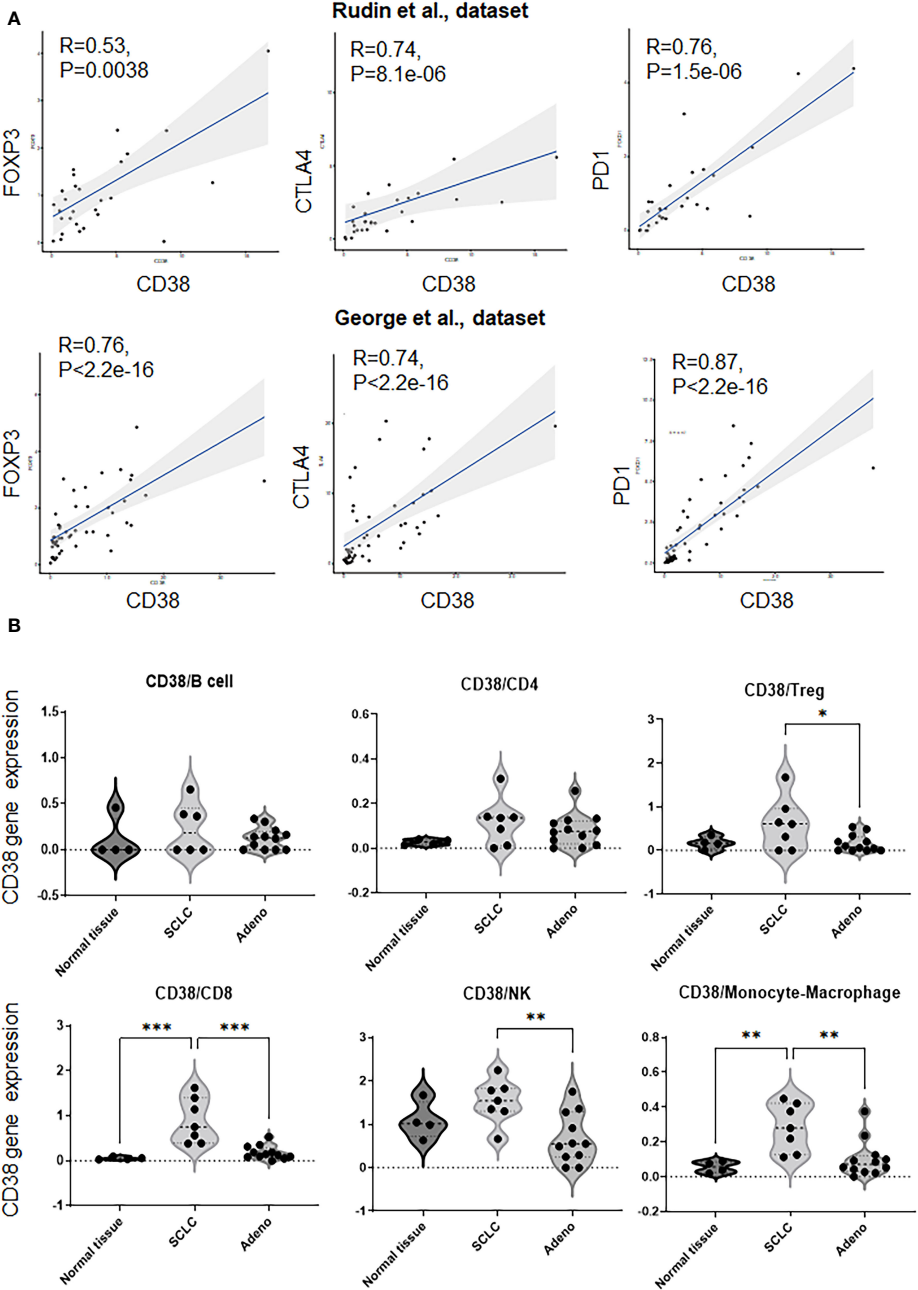
Correlation with CD38 and immune markers in SCLC datasets. **(A)**. Correlation with CD38 and FOXP3, CTLA-4, PD-1. [upper; Rudin et al., dataset (5), bottom; George et al., dataset (6)]. P values are calculated using Spearman rank correlation test. **(B)** Frequency of CD38 [log2 transform with a pseudo-count of 1)] on B cell, CD4^+^ T cell, regulatory T cell, CD8^+^ T cell, and NK cell in a single cell atlas of human SCLC (Treatment naïve patients). p values were calculated by one-way ANOVA and adjusted P values were by Turkey’s test. * *p <*0.0332, ** *p <*0.0021, *** *p <*0.0002.

In both datasets, we discovered a significant correlation between transcript levels of *CD38* with the expression of immunosuppressive markers *FOXP3, CTLA4* and *PD-1* (**Figure 1A**). We further looked into a larger subset of host immune markers and found positive correlation between *CD38* expression and *PD-L1* (**Supplementary Figure S1**).

Therefore, *CD38* expression is correlated with the expression of multiple immune suppressive markers.

### Analysis of CD38 expression on immune cells of SCLC clinical samples as analyzed by single cell RNA sequencing

To evaluate the expression of CD38 on SCLC focusing on immune cells, we next evaluated the expression of CD38 on the immune cells in a single cell RNA sequencing-dataset that we recently reported of human SCLC (7).

Interestingly, we found that frequency of CD38 on CD8^+^ cytotoxic T cells or on monocytes/macrophages was significantly higher in treatment-naïve SCLC samples as compared to normal tissues or treatment-naïve lung adenocarcinoma (**Figure 1B**). In addition, the frequency of CD38 on regulatory T cells or natural killer cells was significantly higher in treatment-naïve SCLC samples as compared to lung adenocarcinoma (**Figure 1B**). However, CD38 did not show significant difference on B cells and CD4^+^ T cells between these subsets (**Figure 1B**).

SCLC is categorized into four major subtypes by relative gene expression of four key transcription regulators defining subtypes: SCLC-A (ASCL1), SCLC-N (NeuroD1), SCLC-P (POU2F3) and SCLC-Y (YAP1) (8). While this dataset had limited representation of SCLC-P and SCLC-Y, we did not observe any significant difference of the CD38 expression in immune cells between SCLC-A and SCLC-N (**Supplementary Figure S2**).

Our data indicates that CD38 is enriched in SCLC, which is associated with an immune suppressed phenotype.

### Anti-PD-L1 treatment increases the expression of CD38 in murine SCLC models

To assess how CD38 expression is modulated by ICB, we utilized pre-clinical of SCLC derived from genetically engineered mouse models (GEMM)with conditional loss of *Rb1, Trp53* (RP)*, Rb1, Trp53* and *p130* (RPP) or *Rb1, Trp53, and MYC^T58A^* (RPM) closely resembling the human disease (9, 10).

**Figure 2:**
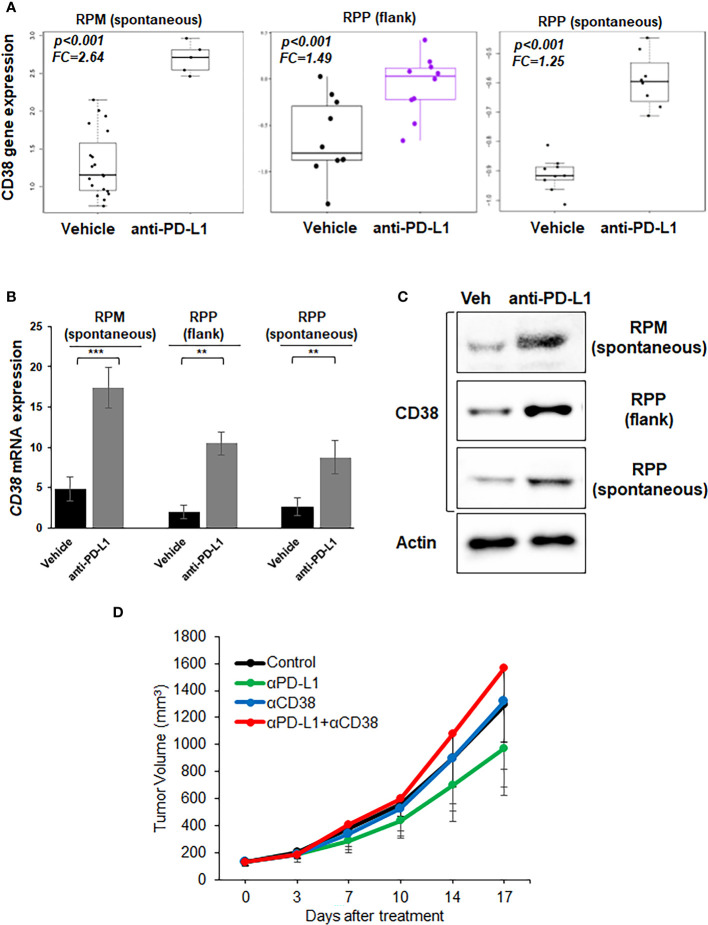
Expression of CD38 by treatment with anti-PD-L1 antibody and anti-tumor effect of anti-CD38 antibody with anti-PD-L1 antibody *in vivo*. **(A)** Boxplots from bulk RNAseq analysis corresponding to the *CD38* expression **(B)** Quantitative mRNA expression of *CD38* by real-time RT-PCR analysis **(C)** Western blot showing expression of CD38 and actin (loading control)- in RPP (flank and orthotopic tumors) and RPM flank tumors, which were treated with vehicle or anti-PD-L1 antibody (300µg/body, once weekly). **(D)** Tumor growth curves (mean ± standard error [SE]) of IgG control, anti-PD-L1 antibody alone (200 µg/body, once weekly), anti-CD38 antibody (300 µg/body, once weekly), and anti-CD38 antibody plus anti-PD-L1 antibody groups in B6129F1 mice injected with RPP cells (SCLC cells with conditional loss of *Trp53, p130*, and *Rb1; n* = 6 in each group). P values were calculated by student-T test.

We observed the highest expression of CD38 RNA in RPM model which has stabilization of *MYC* (**Supplementary Figure S3**). We next treated immunocompetent B6129F1 mice bearing RPP flank tumors and B6FVBF1/J mice bearing flank and lung (spontaneous) RPM tumors with anti-PD-L1 antibody. The tumors were collected for transcriptomic analysis. Bulk RNAseq showed that PD-L1 treatment significantly enhanced *CD38* expression in all three models (**Figure 2A**). The bulk RNAseq data was confirmed by RT-PCR analysis (**Figure 2B**). We confirmed protein expression of CD38 by western blot analysis of tumors from the three models (**Figure 2C**).

Next, we investigated whether CD38 blockade enhanced the anti-tumor immune effect of anti-PD-L1 antibody *in vivo*. CD38 blockade either as a single agent or in combination with anti-PD-L1 did not suppress the tumor growth in the immune competent-mouse subcutaneous RPP model as compared to vehicle control or anti-PD-L1 treatment (**Figure 2D**).

Therefore, collectively our data shows that PD-L1 blockade leads to CD38 upregulation both at RNA and protein level. However, CD38 blockade does not augment the anti-tumor efficacy of PD-L1 blockade in an immunocompetent mouse model of SCLC.

### Analysis of CD38 expression on immune cells post-chemotherapy and -ICB treatment in SCLC clinical samples as analyzed by multi-color flow cytometry

Chemotherapy in combination with immunotherapy is the standard-of-care first line therapy for SCLC. Therefore, to identify the role expression of CD38 post-chemotherapy treatment, we further evaluated the protein expression of CD38 on the cell surface of tumor-infiltrating immune cells in SCLC with clinical samples from treatment-naive tumors, or post-chemotherapy and ICB by multi-color flow cytometry.

**Figure 3:**
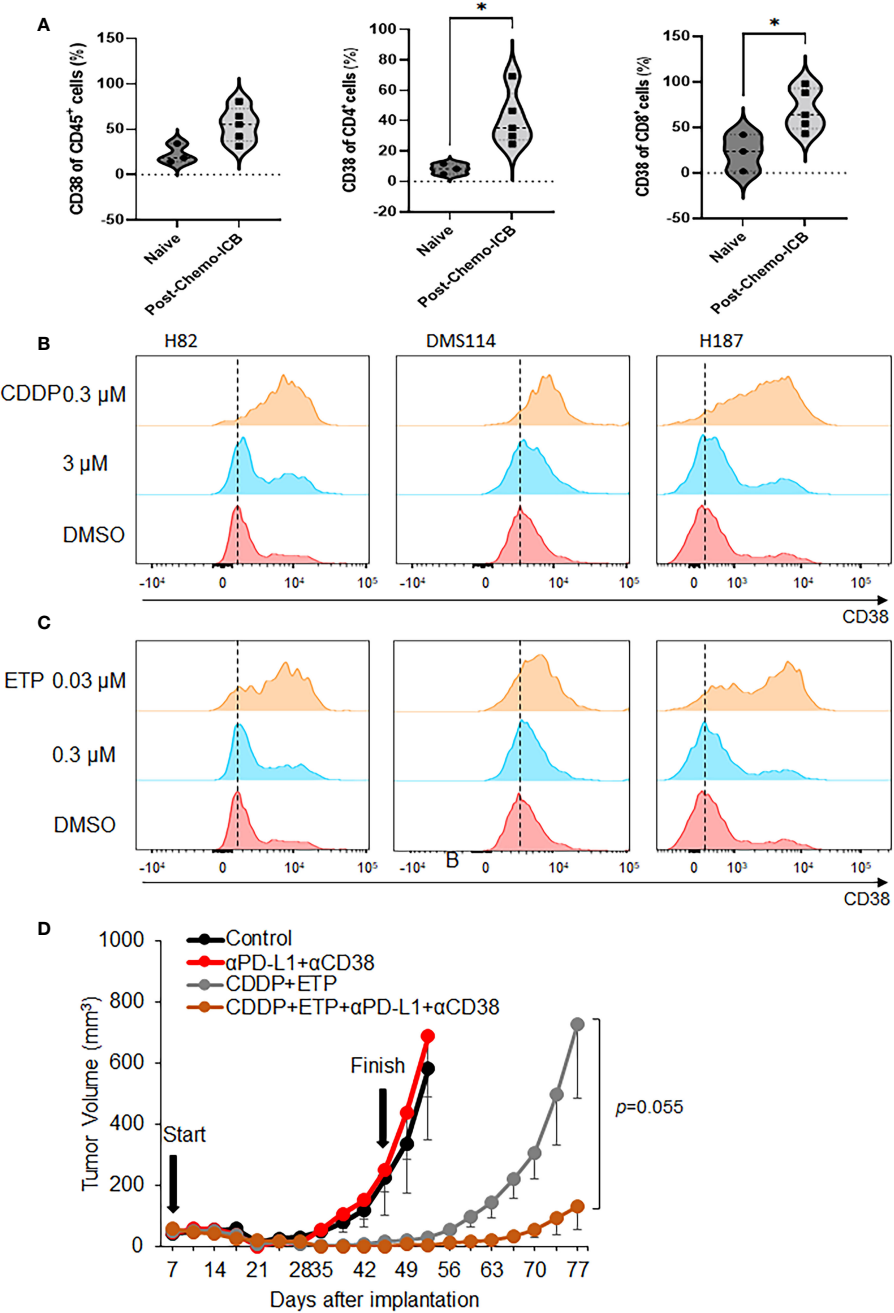
Change of expression of CD38 by chemotherapy and anti-tumor effect of anti-CD38 antibody with anti-PD-L1 antibody and chemotherapy. **(A)** CD38 expression on CD45^+^ total immune cell, CD4^+^ T cell, and CD8^+^ T cell in clinical specimen from the patients with SCLC. **(B, C)** Flow cytometry analysis was performed on H82, DMS114, and H187 cell lines treated with 0.3 or 3 µM cisplatin (upper) and 0.03 or 0.3 µM etoposide for 72 hours. **(D)** Tumor growth curves (mean ± standard error [SE]) of IgG control and vehicle, anti-PD-L1 antibody alone (200 µg/body, once weekly) and anti-CD38 antibody (300 µg/body, once weekly), cisplatin (CDDP) (2mg/kg) and etoposide (ETP) (3mg/kg), and anti-CD38 antibody, anti-PD-L1 antibody, CDDP and ETP groups in B6129F1 mice injected with RPP cells (SCLC cells with conditional loss of *Trp53, p130*, and *Rb1; n* = = 6 in each group). P values were calculated by Mann-Whiteney for **(A)**, * p<0.0332 and student-T test for **(D)**.

The CD38 expression on CD4^+^ T cells and CD8^+^ cytotoxic T cells of post-treatment samples were significantly higher compared to that of treatment-naïve samples. CD38 expression on CD45^+^ total immune cells was tended to be higher in post-treatment compared to pre-treatment group, indicating that the expression of CD38 may be affected by chemotherapy and ICB (**Figure 3A**, **Supplementary Table S1**).

To further confirm our findings, we treated three SCLC cell lines with cisplatin (**Figure 3B**) or etoposide (**Figure 3C**) and measured the cell surface expression of CD38 by flow cytometry. In agreement with the clinical data, treatment with either cisplatin or etoposide enhanced the level of CD38 in all three models.

Together, these findings indicate that chemotherapy treatment induces the expression of CD38 in SCLC and suggest that CD38 induction may be a biomarker of chemotherapy resistance in SCLC.

### Concomitant blockade of CD38 and PD-L1 enhances the efficacy of chemotherapy in SCLC

We next investigated the effects of concomitantly blocking CD38 and PD-L1 in combination with cytotoxic chemo-agents. The RPP subcutaneous model was treated with either vehicle, αPD-L1+αCD38, cisplatin/etoposide, αPD-L1+αCD38+cisplatin/etoposide starting the treatment from early timing. As expected, cisplatin/etoposide treatment significantly delayed in tumor growth recapitulating early responses seen in patients with SCLC (p=0.00033, Student T test, on day 52). Interestingly, combination of cisplatin/etoposide, anti-PD-L1 antibody and anti-CD38 antibody predominantly delayed tumor growth compared to cisplatin and etoposide (p=0.055, Student T test), suggesting that co-inhibition of CD38 and PD-L1 combined with chemo-agents might show anti-tumor effect in SCLC (**Figure 3D**, **Supplementary Figure S4**).

Taken together, co-inhibition of CD38 and PD-L1 enhances the efficacy of cisplatin and etoposide treatment in SCLC.

## Discussion

SCLC exhibits a cold and heterogeneous T-cell infiltration that could potentially lead to ineffective antitumor immune surveillance (11). Furthermore, chemotherapy (cisplatin/carboplatin and etoposide) in addition to anti-PD-L1 antibody improved median progression-free survival by only two months (12, 13). Therefore, we and other groups have investigated strategies to enhance the efficacy of PD-1/PD-L1 blockade with DNA damage response inhibitors (14, 15), LSD1 inhibitor (16), or EZH2 inhibitor (17). Although co-inhibition of CD38 and PD-L1 improves antitumor immune response in NSCLC as previously reported (2), the co-inhibition did not demonstrate anti-tumor effects in a SCLC mouse model in this study. Since CD38 expression was increased by cisplatin or etoposide on SCLC cells *in vitro*, the direct blockade of CD38 on tumor cells starting early timing might suppress the tumor growth.

CD38 is involved in an ectoenzymatic network responsible for generating adenosine (ADO) from different substrates, such as ATP and NAD^+^. The conventional pathway for ADO production includes CD39, which converts ATP to ADP and AMP, and CD73 (ecto-5’-nucleotidase), which converts AMP to ADO. Regulatory T cells commonly express both CD39 and CD73, playing a crucial role in regulatory T cells-mediated immune-regulatory functions. Therefore, CD38 may interact with other transmembrane glycoproteins, participating in diverse cellular processes (18).

Previous papers reported that the roles of CD38 in immunosuppression, chemoresistance and tumor relapse. CD38 suppresses the function of CD8^+^ T-cells through signaling via adenosine receptors, and blocking either CD38 or adenosine receptors represents effective strategies for overcoming resistance (2). In NSCLC cells, the knocking out of CD38 resulted in heightened levels of NAD^+^, subsequently leading to enhanced infiltration of CD8^+^ T cells and a favorable prognosis (19). In addition, clinical data have shown that the elevation of tumor-derived CD38 correlates with poor prognosis in patients with NSCLC. Also, it was found that the enzymatic activity of tumor-expressed CD38 promotes the migration, proliferation, colony formation, and tumor development of lung cancer cells. The *in vivo* experiments consistently demonstrated that inhibiting the enzymatic activity of CD38 or blocking its enzymatic product led to similar inhibitory effects on tumor proliferation and metastasis, comparable to CD38 gene knockout or mutation. Furthermore, at the biochemical level, it was identified that cADPR, a primary hydrolytic product of CD38, induced the opening of TRPM2 iron channels, resulting in intracellular Ca2^+^ influx and subsequent upregulation of NRF2 levels while downregulating KEAP1 expression in NSCLC cells (20).

In this study, we found a significant correlation between transcript levels of *CD38* with the expression of immunosuppressive markers *FOXP3, CTLA4* and *PD-1* in SCLC (**Figure 1A**). In supports of these results, previous preclinical studies have reported the roles of CD38 as immune-modulatory reported. Mice with a knocked-out CD38 gene exhibited an expedited onset of autoimmune diabetes and a compromised development of regulatory T cells (21). CD38-expressing CD8^+^ suppressed the proliferation of CD4^+^ T lymphocytes and alleviated the symptoms of autoimmune encephalomyelitis in mice (22). These findings suggest that CD38 plays a suppressive role in immune regulation, inhibiting the activity of cytotoxic T cells and blocking CD38 concurrently with chemotherapy and anti-PD-L1 antibodies may have activated cancer immunity, while the detailed mechanism remains elusive.

## Limitations of the study

This study has several limitations; the small number and unpaired clinical samples (i.e, pre-treatment and post-treatment tumor samples were collected from different patients) and only one mouse model with subcutaneous tumor was tested. Detailed mechanisms of the CD38 expression induced by chemotherapy and the induction of CD38 by other drugs such as lurbinectedin or irinotecan remain unclear. Our results indicate the role of CD38 in SCLC as a first investigational study. Future studies in large cohort of clinical samples and *in vivo* experiment, which assess tumor tissues using flowcytometry, immunohistochemistry to verify the location of CD38 in tumors, analyze CD38-positive T cells in peripheral blood, and *in vivo* studies, including validation our findings using CD38 knockout model, will be valuable to clarify clinical translational strategies for the potential utilization of concomitant CD38 and PD-L1 blockade with chemotherapy and immunotherapy in SCLC.

## Conclusion

In summary, we investigate the roles of CD38 in SCLC in terms of the immune microenvironment. The expression of CD38 positively correlates the expression of immunosuppressive markers such as FOXP3, CTLA4, PD-1 in SCLC datasets. CD38 on CD8^+^ cytotoxic T cell, NK cell, or regulatory T cell express higher levels of CD38 in SCLC compared to lung adenocarcinoma. Treatment with chemotherapy and ICB enhances the expression CD38 on T cells, suggesting that the addition of CD38 blockade may have the potential to augment the efficacy of chemotherapy and PD-L1 blockade in SCLC.

## Data availability statement

The raw data supporting the conclusions of this article will be made available by the authors, without undue reservation.

## Ethics statement

The animal study was approved by Memorial Sloan Kettering Cancer Center (MSKCC) Animal Care and Use Committee. The study was conducted in accordance with the local legislation and institutional requirements. Written, informed consent was obtained from the patients/participants. Studies involving human participants were approved by the MSKCC IRB.

## Author contributions

HT: Validation, Formal analysis, Data curation, Writing – review & editing, Writing – original draft, Visualization, Methodology, Investigation. SC: Writing – review & editing, Methodology, Formal analysis, Data curation. AC: Data curation, Visualization, Validation, Investigation, Writing – review & editing. JC: Formal analysis, Data curation, Writing – review & editing, Validation. HM: Resources, Writing – review & editing. CR: Funding acquisition, Writing – review & editing, Resources. TS: Writing – original draft, Visualization, Supervision, Project administration, Methodology, Investigation, Conceptualization, Writing – review & editing, Resources, Funding acquisition.

## Supplementary material

Supplementary Figure 1 Gene expression for immune markers showing an increasing trend with increase in CD38 expression. [upper; Rudin et al., dataset (5), bottom; George et al., dataset (6)].

Supplementary Figure 2 Analysis of CD38 related immune system from the data set of SCLC-ATLAS focused on SCLC subtypes. Frequency of CD38 on CD4^+^ T cell, CD8^+^ T cell, regulatory T cell, NK cell, and B cell in a single cell atlas of human SCLC (SCLC-A *vs* SCLC-N). P value was calculated with Mann-Whiteney test.

Supplementary Figure 3 Baseline mRNA expression of *CD38* assessed by real-time RT-PCR in RP, RPP and RPM tumors.

Supplementary Figure 4 Individual tumor growth curves in the two treatment groups: CDDP+ETP (upper) and CDDP+ETP+αPD-L1+αCD38 (n=6) in B6129F1 mice injected with RPP cells (SCLC cells with conditional loss of *Trp53, p130*, and *Rb.*

Supplementary Table 1 Clinical and pathological features of clinical biospecimens.

## References

[1] Quarona V Zaccarello G Chillemi A Brunetti E Singh VK Ferrero E. CD38 and CD157: a long journey from activation markers to multifunctional molecules. Cytom B Clin Cytom. (2013) 84 (4):207–17. doi: 10.1002/cyto.b.21092 23576305

[2] Chen L Diao L Yang Y Yi X Rodriguez BL Li Y. CD38-mediated immunosuppression as a mechanism of tumor cell escape from PD-1/PD-L1 blockade. Cancer Discov. (2018) 8 (9):1156–75. doi: 10.1158/2159-8290.CD-17-1033 PMC6205194 30012853

[3] Antonia SJ Lopez-Martin JA Bendell J Ott PA Taylor M Eder JP. Nivolumab alone and nivolumab plus ipilimumab in recurrent small-cell lung cancer (CheckMate 032): a multicentre, open-label, phase 1/2 trial. Lancet Oncol. (2016) 17 (7):883–95. doi: 10.1016/S1470-2045(16)30098-5 27269741

[4] Ott PA Elez E Hiret S Kim DW Morosky A Saraf S. Pembrolizumab in patients with extensive-stage small-cell lung cancer: results from the phase ib KEYNOTE-028 study. J Clin Oncol. (2017) 35 (34):3823–9. doi: 10.1200/JCO.2017.72.5069 28813164

[5] Rudin CM Durinck S Stawiski EW Poirier JT Modrusan Z Shames DS. Comprehensive genomic analysis identifies SOX2 as a frequently amplified gene in small-cell lung cancer. Nat Genet. (2012) 44 (10):1111–6. doi: 10.1038/ng.2405 PMC3557461 22941189

[6] George J Lim JS Jang SJ Cun Y Ozretić L Kong G. Comprehensive genomic profiles of small cell lung cancer. Nature. (2015) 524 (7563):47–53. doi: 10.1038/nature14664 26168399 PMC4861069

[7] Chan JM Quintanal-Villalonga Á Gao VR Xie Y Allaj V Chaudhary O. Signatures of plasticity, metastasis, and immunosuppression in an atlas of human small cell lung cancer. Cancer Cell. (2021) 39 (11):1479–96.e18. doi: 10.1016/j.ccell.2021.09.008 34653364 PMC8628860

[8] Rudin CM Poirier JT Byers LA Dive C Dowlati A George J. Molecular subtypes of small cell lung cancer: a synthesis of human and mouse model data. Nat Rev Cancer. (2019) 19 (5):289–97. doi: 10.1038/s41568-019-0133-9 PMC6538259 30926931

[9] Schaffer BE Park KS Yiu G Conklin JF Lin C Burkhart DL. Loss of p130 accelerates tumor development in a mouse model for human small-cell lung carcinoma. Cancer Res. (2010) 70 (10):3877–83. doi: 10.1158/0008-5472.CAN-09-4228 PMC2873158 20406986

[10] Mollaoglu G Guthrie MR Böhm S Brägelmann J Can I Ballieu PM. MYC drives progression of small cell lung cancer to a variant neuroendocrine subtype with vulnerability to aurora kinase inhibition. Cancer Cell. (2017) 31 (2):270–85. doi: 10.1016/j.ccell.2016.12.005 PMC5310991 28089889

[11] Chen M Chen R Jin Y Li J Hu X Zhang J. Cold and heterogeneous T cell repertoire is associated with copy number aberrations and loss of immune genes in small-cell lung cancer. Nat Commun. (2021) 12 (1):6655. doi: 10.1038/s41467-021-26821-8 34789716 PMC8599854

[12] Horn L Mansfield AS Szczęsna A Havel L Krzakowski M Hochmair MJ. First-line atezolizumab plus chemotherapy in extensive-stage small-cell lung cancer. N Engl J Med. (2018) 379 (23):2220–9. doi: 10.1056/NEJMoa1809064 30280641

[13] Goldman JW Dvorkin M Chen Y Reinmuth N Hotta K Trukhin D. Durvalumab, with or without tremelimumab, plus platinum-etoposide versus platinum-etoposide alone in first-line treatment of extensive-stage small-cell lung cancer (CASPIAN): updated results from a randomised, controlled, open-label, phase 3 trial. Lancet Oncol. (2021) 22 (1):51–65. doi: 10.1016/S1470-2045(20)30539-8 33285097

[14] Sen T Rodriguez BL Chen L Corte CMD Morikawa N Fujimoto J. Targeting DNA damage response promotes antitumor immunity through STING-mediated T-cell activation in small cell lung cancer. Cancer Discov. (2019) 9 (5):646–61. doi: 10.1158/2159-8290.CD-18-1020 PMC6563834 30777870

[15] Taniguchi H Caeser R Chavan SS Zhan YA Chow A Manoj P. WEE1 inhibition enhances the antitumor immune response to PD-L1 blockade by the concomitant activation of STING and STAT1 pathways in SCLC. Cell Rep. (2022) 39 (7):110814. doi: 10.1016/j.celrep.2022.110814 35584676 PMC9449677

[16] Nguyen EM Taniguchi H Chan JM Zhan YA Chen X Qiu J. Targeting lysine-specific demethylase 1 rescues major histocompatibility complex class I antigen presentation and overcomes programmed death-ligand 1 blockade resistance in SCLC. J Thorac Oncol. (2022) 17 (8):1014–31. doi: 10.1016/j.jtho.2022.05.014 PMC9357096 35691495

[17] Mahadevan NR Knelson EH Wolff JO Vajdi A Saigí M Campisi M. Intrinsic immunogenicity of small cell lung carcinoma revealed by its cellular plasticity. Cancer Discov. (2021) 11 (8):1952–69. doi: 10.1158/2159-8290.CD-20-0913 PMC8338750 33707236

[18] Morandi F Airoldi I Marimpietri D Bracci C Faini AC Gramignoli R. CD38, a receptor with multifunctional activities: from modulatory functions on regulatory cell subsets and extracellular vesicles, to a target for therapeutic strategies. Cells. (2019) 8(12):1527. doi: 10.3390/cells8121527 31783629 PMC6953043

[19] Bu X Kato J Hong JA Merino MJ Schrump DS Lund FE. CD38 knockout suppresses tumorigenesis in mice and clonogenic growth of human lung cancer cells. Carcinogenesis. (2018) 39 (2):242–51. doi: 10.1093/carcin/bgx137 PMC5862338 29228209

[20] Gao L Liu Y Du X Ma S Ge M Tang H. The intrinsic role and mechanism of tumor expressed-CD38 on lung adenocarcinoma progression. Cell Death Dis. (2021) 12 (7):680. doi: 10.1038/s41419-021-03968-2 34226519 PMC8256983

[21] Chen J Chen YG Reifsnyder PC Schott WH Lee CH Osborne M. Targeted disruption of CD38 accelerates autoimmune diabetes in NOD/Lt mice by enhancing autoimmunity in an ADP-ribosyltransferase 2-dependent fashion. J Immunol. (2006) 176 (8):4590–9. doi: 10.4049/jimmunol.176.8.4590 16585549

[22] Bahri R Bollinger A Bollinger T Orinska Z Bulfone-Paus S. Ectonucleotidase CD38 demarcates regulatory, memory-like CD8+ T cells with IFN-γ-mediated suppressor activities. PloS One. (2012) 7 (9):e45234. doi: 10.1371/journal.pone.0045234 23028866 PMC3444472

