# Supplementary material for: Role of CD38 in anti-tumor immunity of small cell lung cancer
Source: Front Immunol. 2024 Mar 12;15:1348982. doi: 10.3389/fimmu.2024.1348982 (PMC10963403; doi:10.3389/fimmu.2024.1348982)
Supplement: Supplementary Figure 1 — Gene expression for immune markers showing an increasing trend with increase in CD38 expression. [upper; Rudin et al., dataset (5), bottom; George et al., dataset (6)]. [file Presentation_1.pptx]

## Slide 1
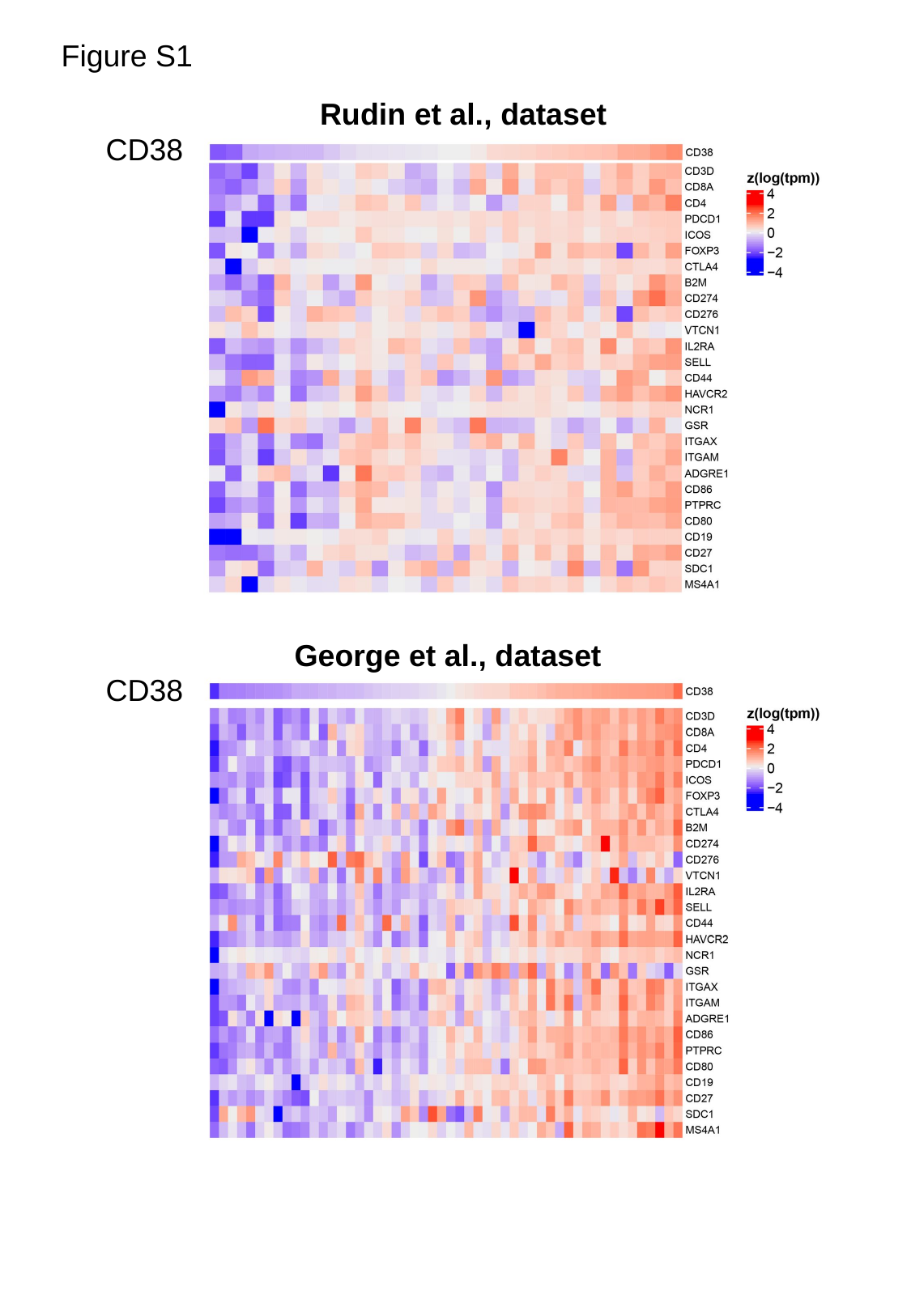

Figure S1
Rudin et al., dataset
CD38
CD38
George et al., dataset
CD38

## Slide 2
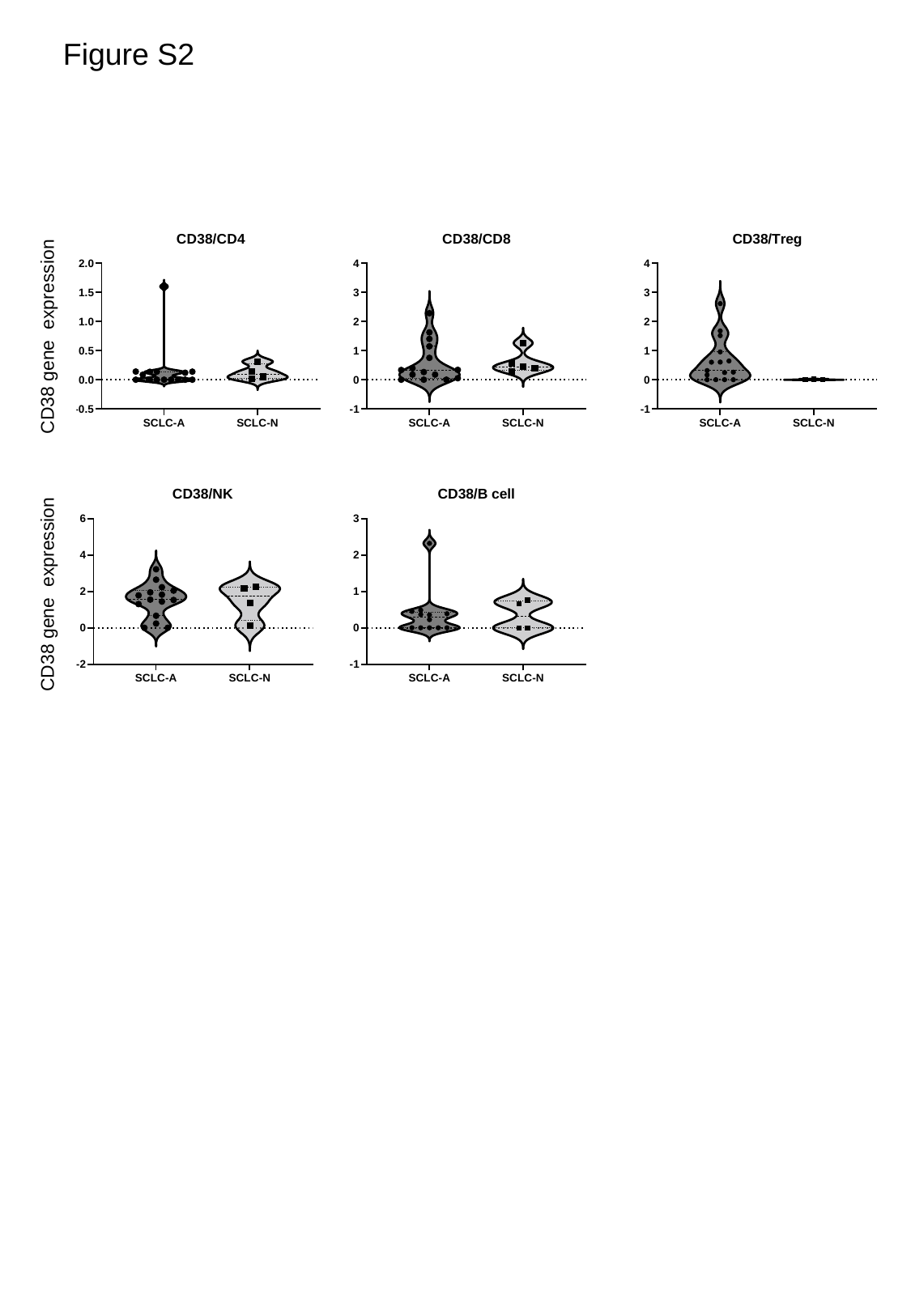

Figure S2
CD38 gene expression
CD38 gene expression

## Slide 3
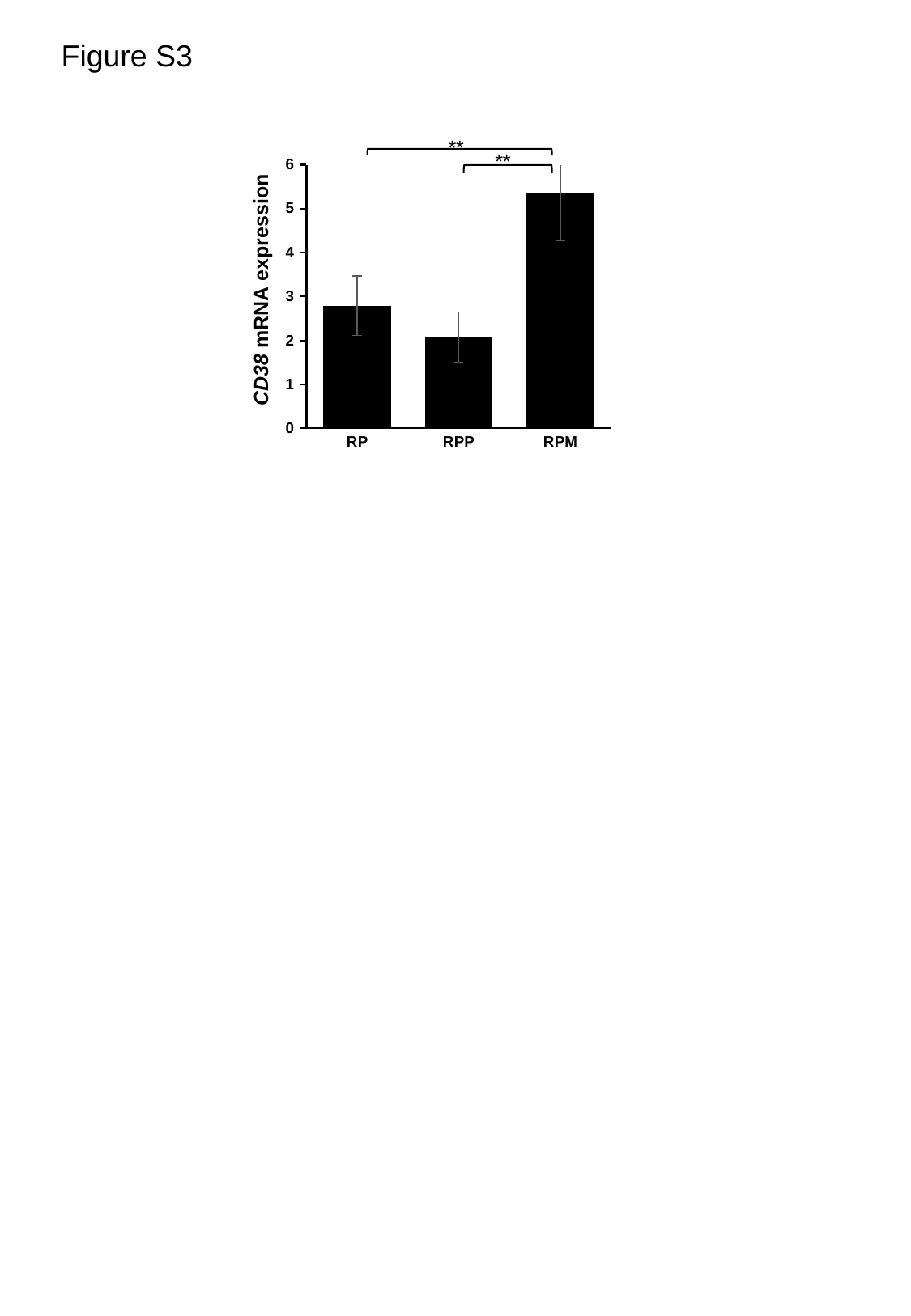

Figure S3
**
### Chart
| Category | |
|---|---|
| RP | 2.7894 |
| RPP | 2.0748 |
| RPM | 5.3698 |
**
CD38 mRNA expression

## Slide 4
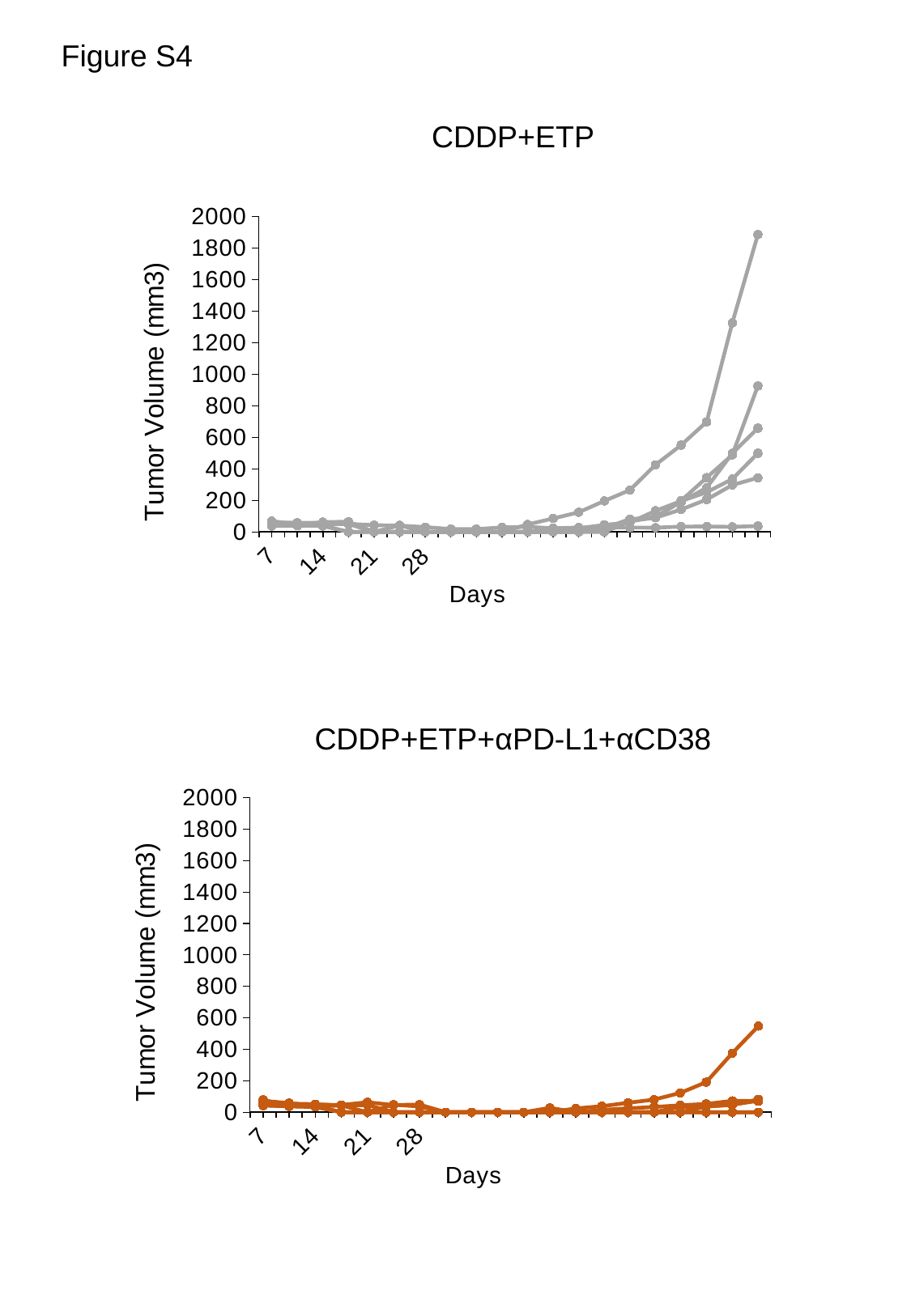

Figure S4
CDDP+ETP
### Chart
| Category | | | | | | |
|---|---|---|---|---|---|---|
| 7 | 46.464000000000006 | 37.6 | 68.479 | 40.572 | 44.528 | 59.903999999999996 |
| | 44.982 | 37.926 | 48.400000000000006 | 48.074 | 44.1 | 58.18999999999999 |
| 14 | 62.208 | 40.678000000000004 | 53.662499999999994 | 47.43200000000001 | 37.6 | 57.599999999999994 |
| | 64.512 | 0.0 | 50.42999999999999 | 50.78399999999999 | 0.0 | 57.132 |
| 21 | 0.0 | 0.0 | 0.0 | 42.526999999999994 | 0.0 | 0.0 |
| | 0.0 | 0.0 | 0.0 | 39.753499999999995 | 0.0 | 41.45400000000001 |
| 28 | 0.0 | 0.0 | 0.0 | 30.419999999999998 | 0.0 | 0.0 |
| 35 | 0.0 | 0.0 | 0.0 | 18.0 | 0.0 | 0.0 |
| | 0.0 | 0.0 | 0.0 | 17.549999999999997 | 0.0 | 0.0 |
| 42 | 0.0 | 0.0 | 0.0 | 28.749000000000002 | 0.0 | 0.0 |
| | 47.14949999999999 | 0.0 | 0.0 | 32.0 | 0.0 | 0.0 |
| 49 | 85.176 | 0.0 | 0.0 | 23.275 | 0.0 | 17.549999999999997 |
| | 124.468 | 0.0 | 0.0 | 27.380000000000003 | 0.0 | 23.119999999999997 |
| 56 | 196.51999999999998 | 27.216 | 0.0 | 26.950000000000003 | 23.4135 | 44.1 |
| | 265.586 | 69.11999999999999 | 65.664 | 27.380000000000003 | 81.25 | 60.01600000000001 |
| 63 | 425.9200000000001 | 89.888 | 97.34400000000002 | 26.6955 | 91.25 | 134.01850000000002 |
| | 550.525 | 142.228 | 189.58399999999997 | 33.6 | 198.57500000000002 | 196.01999999999998 |
| 70 | 698.7684999999999 | 205.8 | 280.845 | 34.4 | 344.45000000000005 | 252.04999999999998 |
| | 1325.646 | 297.6 | 498.6769999999999 | 32.0 | 488.57899999999995 | 334.98850000000004 |
| 77 | 1886.2845 | 343.1875 | 657.9644999999999 | 36.98199999999999 | 925.75 | 499.376 |CDDP+ETP+αPD-L1+αCD38
### Chart
| Category | | | | | | |
|---|---|---|---|---|---|---|
| 7 | 62.208 | 44.8 | 78.73200000000001 | 72.5 | 51.304 | 42.4 |
| | 45.3005 | 37.2645 | 37.926 | 57.599999999999994 | 54.675000000000004 | 37.6 |
| 14 | 35.2 | 32.856 | 36.1 | 40.34399999999999 | 50.8475 | 48.074 |
| | 43.2 | 0.0 | 0.0 | 0.0 | 42.336000000000006 | 45.86400000000001 |
| 21 | 0.0 | 0.0 | 0.0 | 0.0 | 45.86400000000001 | 63.01439999999999 |
| | 47.62800000000001 | 0.0 | 0.0 | 0.0 | 0.0 | 45.6 |
| 28 | 36.504 | 0.0 | 0.0 | 0.0 | 0.0 | 48.074 |
| 35 | 0.0 | 0.0 | 0.0 | 0.0 | 0.0 | 0.0 |
| | 0.0 | 0.0 | 0.0 | 0.0 | 0.0 | 0.0 |
| 42 | 0.0 | 0.0 | 0.0 | 0.0 | 0.0 | 0.0 |
| | 0.0 | 0.0 | 0.0 | 0.0 | 0.0 | 0.0 |
| 49 | 0.0 | 0.0 | 0.0 | 0.0 | 0.0 | 27.380000000000003 |
| | 0.0 | 0.0 | 0.0 | 23.8875 | 0.0 | 0.0 |
| 56 | 14.400000000000002 | 0.0 | 0.0 | 38.025 | 0.0 | 0.0 |
| | 24.5 | 0.0 | 0.0 | 59.64300000000001 | 0.0 | 0.0 |
| 63 | 33.048 | 0.0 | 0.0 | 80.0565 | 0.0 | 0.0 |
| | 44.0 | 34.225 | 0.0 | 122.39999999999999 | 0.0 | 0.0 |
| 70 | 52.27200000000001 | 36.1 | 0.0 | 191.1 | 0.0 | 36.8 |
| | 72.03000000000002 | 47.9115 | 0.0 | 375.584 | 0.0 | 45.86400000000001 |
| 77 | 70.688 | 72.89999999999999 | 0.0 | 547.4280000000001 | 0.0 | 81.93149999999999 |
